# Efficacy comparison of Kirschner-wire tension band and anchor loop plate in treatment of olecranon fracture

**DOI:** 10.3389/fbioe.2023.1203244

**Published:** 2023-09-01

**Authors:** Bing Du, Yu Su, Teng Ma, Shuai Ji, Yao Lu, Kun Zhang, Zhong Li, Ming Li

**Affiliations:** Xi’an Honghui Hospital, Xi’an Jiaotong University, Xi’an, China

**Keywords:** olecranon fracture, anchor loop plate, tension band, internal fixation, finite element analysis

## Abstract

**Objective:** This study aimed to introduce a new surgical method for the fixation of olecranon fractures, and to compare the biomechanical stability and clinical efficacy of Kirschner wire tension band and anchor loop plate (ALP) in the treatment of olecranon fractures.

**Methods:** A finite element model was established to analyze the mechanical properties of Kirschner wire tension and anchor loop plate fixation for olecranon fracture. The clinical data of 53 patients with olecranon fractures admitted to our hospital from March 2016 to October 2021 were retrospectively analyzed. Among them, 22 cases were fixed with an anchor loop plate (ALP group), and 31 patients were fixed with the Kirschner wire tension band technique. By reviewing the medical records and follow-up results, the final elbow mobility, secondary surgery, postoperative complications and elbow function recovery Mayo score and DASH score were compared between the two groups.

**Results:** The biomechanical analysis of the finite element model showed that under the load of 120 N, the maximum displacement of the Kirschner wire group was 1.09 times that of the ALP group, the maximum stress of the Kirschner wire group was 1.33 times that of the ALP group, and the maximum stress of the olecranon proximal bone of the Kirschner wire group was 2.17 times that of the ALP group. Under the load of 200 N, the maximum displacement of the Kirschner wire group was 1.19 times that of the ALP group. The overall maximum stress of the Kirschner wire group was 1.59 times that of the ALP group, and the maximum stress of the proximal olecranon bone of the Kirschner wire group was 1.99 times that of the ALP group. The average follow-up time of the Kirschner wire and anchor loop plate groups was similar (*p* > 0.05). The average age of the two groups was identical (*p* > 0.05). The final elbow mobility in the anchor loop plate group was significantly greater than in the Kirschner wire group (*p* < 0.05). The Mayo score of the anchor loop plate group was substantially higher than that of the Kirschner wire group at 3 and 12 months after operation (*p* < 0.05), and the DASH score was significantly lower than that of the Kirschner wire group (*p* < 0.05). Postoperative complications in the two groups: 1 case (4.5%) in the anchor loop plate group had difficulties with internal fixation stimulation, and no infection occurred; in the Kirschner wire group, 5 cases (16.1%) had complications of internal fixation stimulation, and 1 patient (3.2%) had an infection.

**Conclusion:** The model of olecranon fracture fixed by anchor loop plate and Kirschner wire tension technique was tested under 120 and 200 N tension, and no damage was found, indicating that the newly designed anchor loop plate was safe in mechanical structure. The biomechanical stability of the anchor plate technique is more stable, so it is not easy to have postoperative complications such as fracture block cutting and internal fixation failure. And the secondary operation rate and elbow function have better results. This technique is an effective method for the treatment of olecranon fractures.

## 1 Background

Olecranon fracture is a common elbow injury, mostly in adults, accounting for about 10% of adult upper limb fractures (Rommens et al., 2004; Wiegand et al., 2012). Both direct and indirect violence can cause olecranon fractures. The olecranon and the coronoid process form a semilunar notch-like joint of the proximal ulna, which maintains the stability of the flexion and extension of the elbow joint with the humeral trochlea. After the olecranon fracture occurs, the fracture ends can lead to more significant displacements due to the traction of the surrounding muscles and ligaments (Ali et al., 2017). If not treated in time, it can easily cause severe complications of elbow osteoarthritis or stiffness (Fournet et al., 2018). Olecranon fracture is an intra-articular fracture. Not only it is necessary to perform the anatomical reduction of the articular surface to avoid the occurrence of traumatic arthritis, but also it requires strong fixation to enable patients to perform functional exercises early and avoid elbow stiffness. The Kirschner wire tension band technique has been used as the preferred method for the fixation of an olecranon fracture. It is simple to operate, does not require too much exposure to the stripped soft tissue, and has a good fixation effect. However, the fixation effect of tension band technique applied to olecranon fracture is only applicable to the active extension position of elbow joint. When the humerus bends vertically downward and the forearm actively, it will not lead to load at the fracture end (Brink et al., 2013). Therefore, the compression effect of the fracture end caused by the tension band during functional exercise is reduced in the olecranon. The report on the treatment of olecranon fractures with Kirschner wire tension band found that the application of this technique in the treatment of olecranon fractures has related complications such as internal fixation failure, needle withdrawal, nonunion fracture, internal fixation soft tissue stimulation, and ulnar nerve injury (Gordon et al., 2006; Powell et al., 2019). Scholars have recently applied steel plates to treating olecranon fractures and achieved satisfactory results (Gathen et al., 2020). Although plate fixation has achieved particular success, it is worth noting that the reoperation rate of different plate fixation schemes varies greatly. The probability of reoperation after plate fixation is as high as 15.6% due to soft tissue stimulation of the implant (Bouchard et al., 2022). This complication may be related to the excessive volume of the steel plate, so a surgical technique of anchor loop plate combined with steel wire binding was designed. This technique can firmly fix olecranon fractures, allow patients to perform an early functional exercise, reduce the occurrence of elbow stiffness, and has the advantage of small volume, of what reduces the complications of internal fixation soft tissue stimulation. This study used clinical research and finite element model biomechanical analysis to compare the clinical efficacy and biomechanical characteristics of the anchor loop plate and the Kirschner wire tension band technique in the fixation of an olecranon fracture.

## 2 Materials and methods

### 2.1 Finite element analysis: collection of imaging data

The Ethics Committee of Red Cross Hospital Affiliated to Xi’an Jiaotong University approved this study (No. 202305002). A 30-year-old male patient with olecranon fracture (informed consent and voluntary informed consent) weighing 75 kg was selected. X-ray examination showed that the olecranon bone of the healthy side of the patient had no lesions, and the bone was good. A Spiral CT scan was performed. The scanning target was the healthy olecranon bone of the patient. The scanning length ranged from 5 cm proximal to 20 cm distal to the olecranon. The scanning condition was 120 k V, 155 m A, and the scanning layer thickness was 1 mm. The scanning information was collected and processed, and the continuous image data was saved in DICOM format.

### 2.2 Finite element model of olecranon bone construction

The data were imported into Mimics15.0 software, and a rough model of olecranon bone was established by threshold segmentation and region growth in the software. The obtained data model is imported into Geomagic 2017 software. The model is subdivided into triangular patches, noise reduction and smoothing. The three-dimensional model of cancellous bone and cortical bone of olecranon was constructed by precise surface treatment. The above model was imported into Solidworks185 software to simulate the solid reconstruction of olecranon fracture, and the fracture line was intercepted at the proximal end of olecranon. Both models have homogeneous and linear isotropic material characteristics. In order to ensure the reliability of the research conclusions, we conducted a convergence study. At a unit size of 1 mm, after a tolerance analysis of 95% of the stress results, no stress singularity was found. The experiment was divided into 2 groups: A. Kirschner wire group: 2 Kirschner wires were inserted obliquely into the olecranon of the ulna to the ulnar shaft, tied with a steel wire “8”, and the distal end was hit with a steel wire into the cortical bone. B. Anchor loop plate (ALP) group: The miniplate was bent and attached to the surface of the olecranon bone, and then fixed with 5 screws and 1 steel wire. The silk thread was bundled with “8”, and penetrated the cortical bone near the third screw from the proximal end to the distal end. The contact relationship in both research models is defined as frictional contact.

### 2.3 Volume mesh generation

**FIGURE 1:**
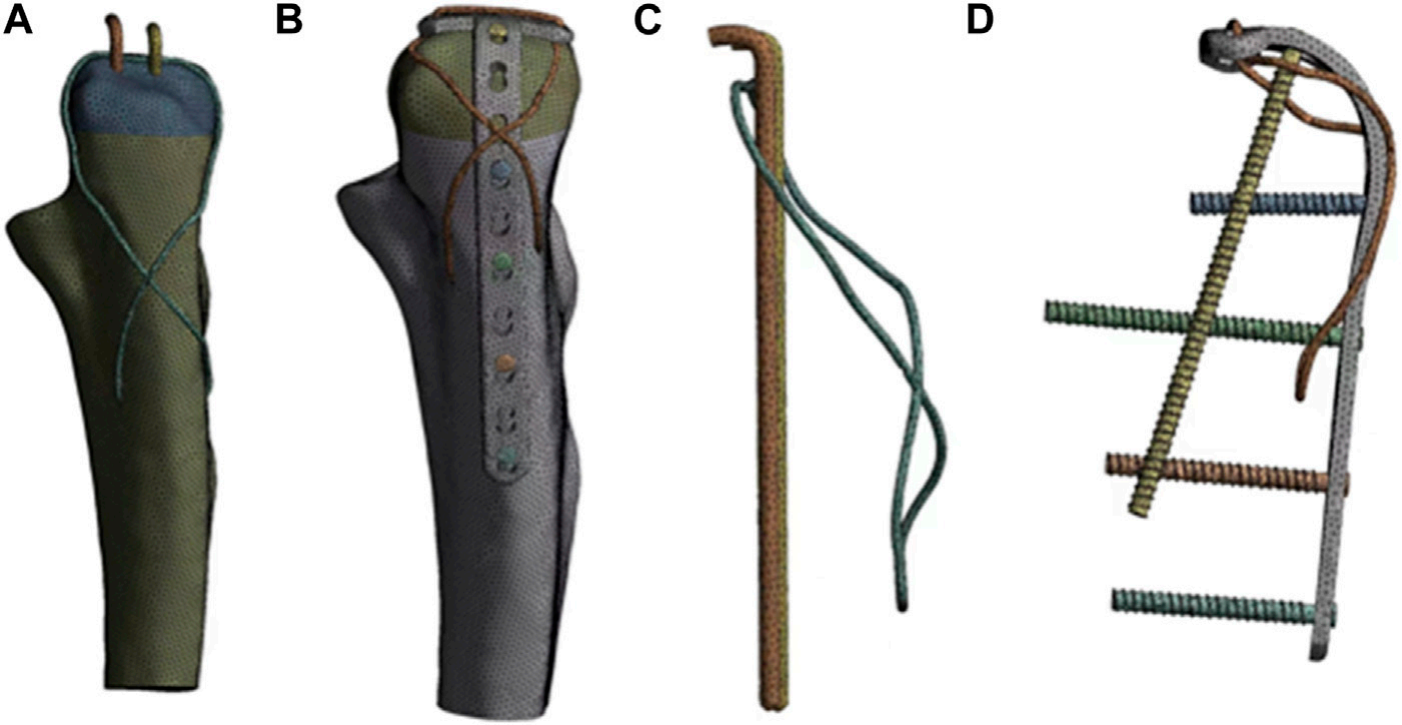
Model after meshing. In the figure, **(A, B)** represent the finite element model of the internal fixation and the whole olecranon fracture after meshing, and **(C, D)** represent the internal fixation model after meshing.

The finite element model was constructed by linear tetrahedron (Figure 1). In this study; there are 706,152 Elements and 1,035,925 nodes in the Kirschner wire group model after meshing. The AP group model has 838,540 Elements and 1,215,467 nodes. The specific values are shown in Table 1.

**TABLE 1:** Number of nodes and elements in finite element model.

Finite element model	Nodes	Elements
A group (K-wire)	1,035,925	706,152
B group (ALP)	1,215,467	838,540

### 2.4 Assignment of material properties

The relevant material properties of the finite element model of olecranon fracture were set up, and a three-dimensional finite element model similar to the actual model in terms of material parameters and mechanical behavior was established. According to the actual data of clinical internal fixation device, two kinds of internal fixation structures involved in this study were constructed by Solidworks software, and the material properties of olecranon cortical bone, olecranon cancellous bone, Kirschner wire, steel wire and titanium alloy (plate and screw) were set (Civan et al., 2021; Kim et al., 2021). The specific parameters are shown in Table 2, the three-dimensional model and internal fixation model of olecranon bone are assembled in SolidWorks software.

**TABLE 2:** Model material parameters.

Name of the material	Elastic modulus (MPa)	Poisson ratio
Cortical bone	1,000	0.30
Cancellous bone	207	0.30
Steel wire	210,000	0.29
Kirschner wire	210,000	0.30
Titanium alloy	110,000	0.30

### 2.5 Constraint and loading

**FIGURE 2:**
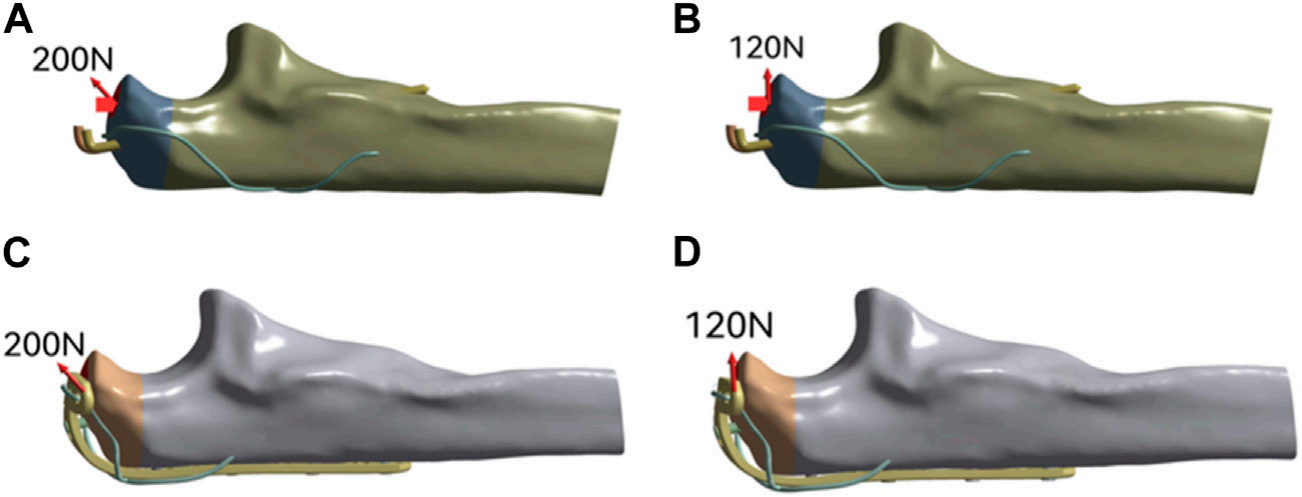
The schematic diagram of load loading of two fixed modes model.

The three-dimensional finite element models of two groups of internal fixation were fixed in the olecranon fracture model. Within 1 s, 120 N force was applied to the 90° direction of the surface of the olecranon fracture block, and 200 N force was applied to the 45° direction (Civan et al., 2021). The magnitude and direction of the loading force are shown in Figure 2.

### 2.6 Comparison of mechanical stability

In this study, the mechanical stability of the two groups of fixation methods refers to the difference in the size of the overall deformation of the two groups of fixation methods, the force of the internal fixation and the force of the fracture block after receiving the load of the same position, size and direction. 1) The overall deformation reflects the overall stability after the fracture end is fixed; 2) The stress of internal fixation reflects the risk of internal fixation failure after fracture fixation. Generally speaking, the greater the local stress, the greater the possibility of internal fixation failure. 3) The more concentrated the force of the olecranon bone, the greater the force, the more likely the olecranon bone is cut by the fixator.

### 2.7 Clinical data

From March 2016 to October 2021, 53 patients with olecranon fractures were included in this study. All patients were divided into the Kirschner wire tension band and anchor loop plate groups by different fracture fixation methods. The hospital ethics committee has approved this experiment, and all patients have signed the informed consent. The inclusion criteria are 1. CT or X-ray diagnosis of olecranon fracture; 2. Using Kirschner wire tension band fixation and anchor loop plate fixation; 3. All operations were completed within 1 week after injury; 4. Patients informed consent and complete clinical data. The exclusion criteria are 1. Patients with other elbow joint fractures; 2. Patients with multiple fractures; 3. Combined with severe ligament injury requiring surgical treatment; 4. Patients with a history of surgery around the elbow; 5. Lost follow-up or follow-up less than 12 months. The patients included in this study were all patients with olecranon fracture Mayo type II. The detailed fracture classification and number of the two groups of patients are shown in Table 3.

**TABLE 3:** Types and number of olecranon fractures (Mayo classification).

	ⅡA	ⅡB
ALP group (n)	20	2
K-wire group (n)	31	0

### 2.8 Surgical procedure

#### 2.8.1 Anchor loop plate group

**FIGURE 3:**
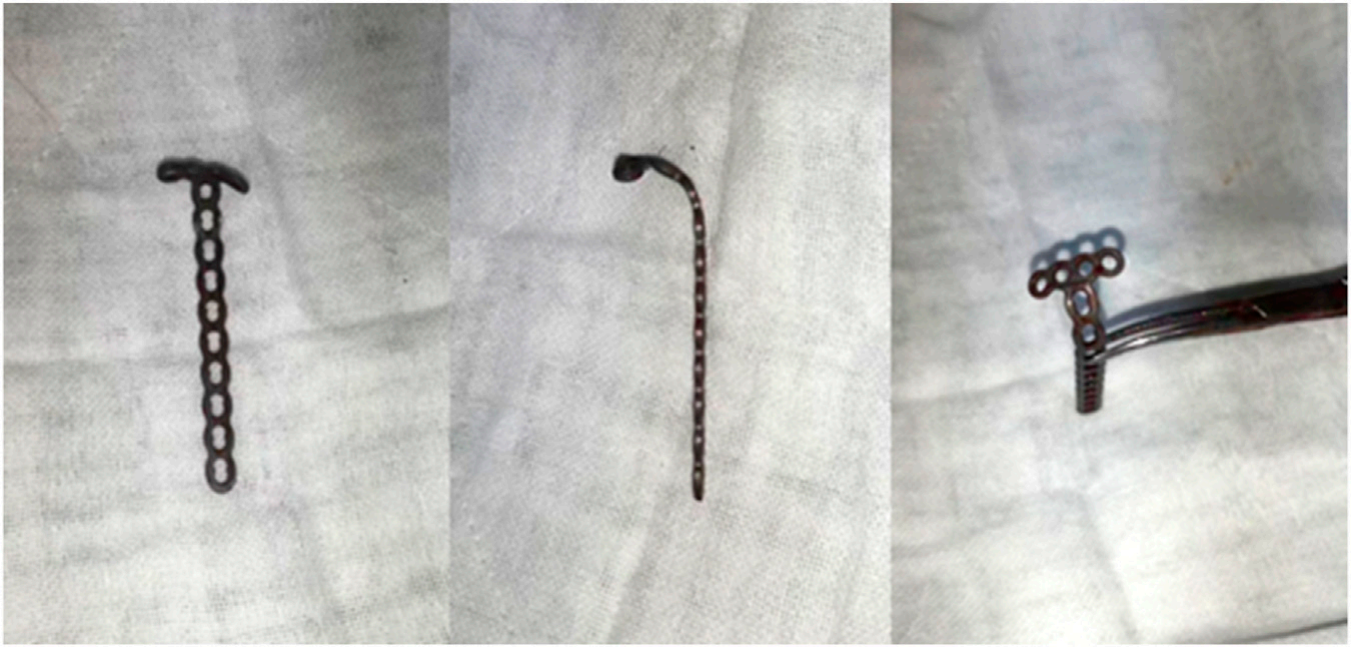
Microplate formed by bending.

The main body of the anchor loop plate used in this technology is formed by bending the microplate. In the process of bending the bone plate, it should be noted that the angle of bending should be attached to the anatomical structure of the olecranon as much as possible. The short arm of the bone plate is not flat but has a certain inclination angle. (Figure 3. Tianjin Zhengtian Company.).

**FIGURE 4:**
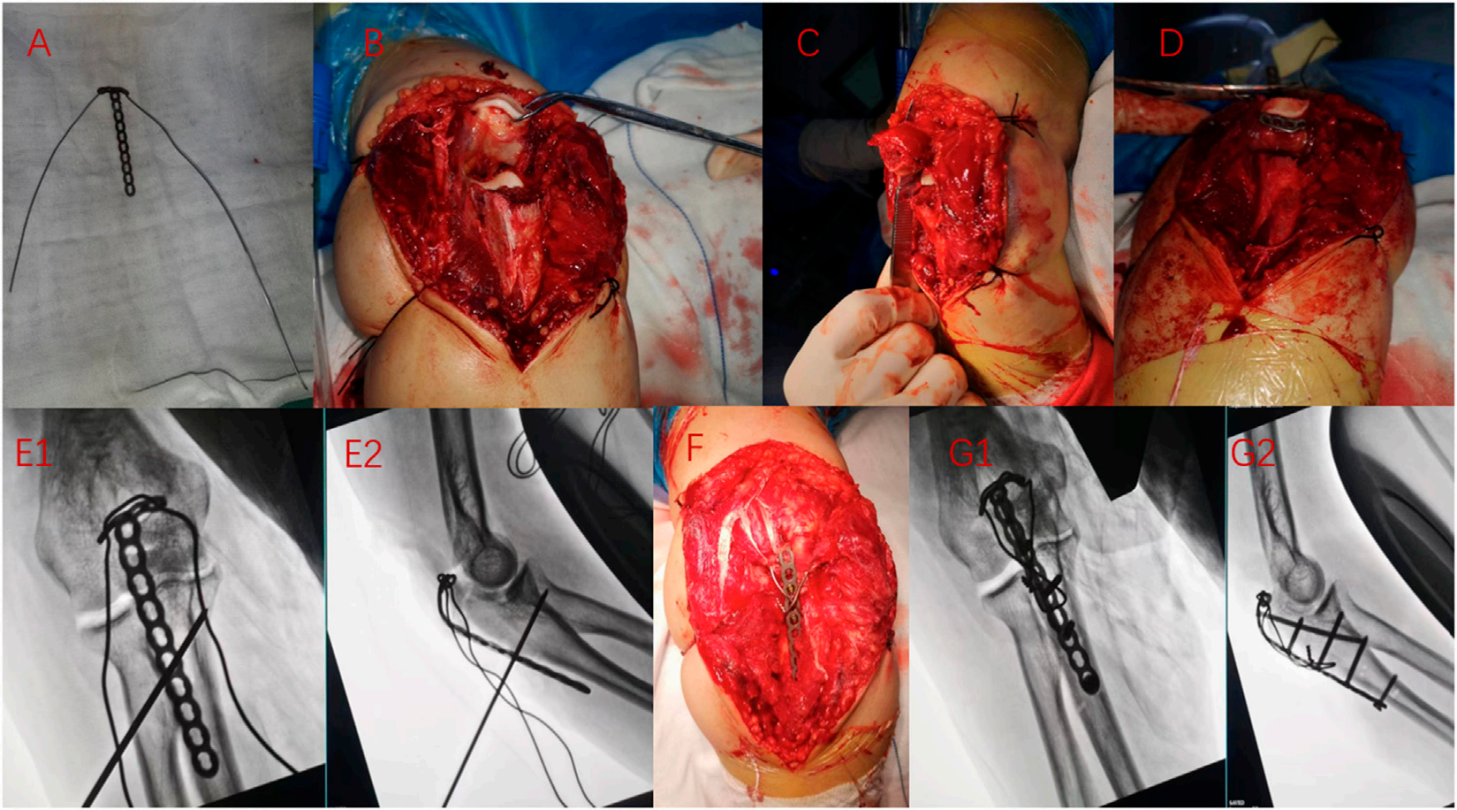
Intraoperative situation of anchor loop plate in treating olecranon fracture. **(A)** Bending micro steel plate, connecting steel wire; **(B, C)** Expose the fracture position, cut a small hole near the olecranon insertion of the triceps tendon; **(D)** Pierce the steel plate through a small hole from the inside out so that the short arm is suspended at the proximal end of the olecranon; **(E1,E2)** Drag the fracture block reduction, Kirschner wire temporary fixation; **(F)** Fix screw, binding wire; **(G1,G2)** Finally, intraoperative X-ray examination.

After adequate anesthesia, the patient was placed in a lateral position, routinely disinfected, covered with sterile surgical towels, and placed a tourniquet near the upper arm. The olecranon fracture was exposed through the posterior approach, the elbow joint cavity was washed, and the blood stasis around the joint and the fracture end was cleaned to confirm the fracture type. We use the anchor loop plate and the steel wire for fixation. After bending the anchor loop plate, the steel wire or titanium cable was inserted into the proximal plate hole to make a “cage lock” anchor loop plate (Figure 4A). A channel was cut near the olecranon insertion of the triceps tendon with a scalpel (Figures 4B, C). The long arm of the anchor loop plate was penetrated by the triceps tendon, and its short arm was hung at the proximal end of the olecranon bone block, so that the olecranon fracture block together with the triceps tendon was considered as a whole (Figure 4D). The long arm was dragged to the distal end of the olecranon for reduction, and the temporary reduction state was maintained with Kirschner wire after reduction (Figure 4E). Finally, a transverse hole was drilled at the distal fracture block, through the steel wire to form an 8-shaped tension band and tighten the steel wire to complete the compression of the fracture end, and then the fracture position was fixed with screws (Figure 4F). The passive motion of the elbow joint is then performed to ensure normal joint motion and stable fixation. C-arm fluoroscopy was used to check the quality of fracture reduction and fixation (Figure 4G), followed by drainage tube placement and wound closure.

#### 2.8.2 K-wire group

The anesthesia and fracture exposure methods were the same as those in the anchor loop plate group, according to the traditional Kirschner wire tension band fixed olecranon fracture standard for internal fixation surgery.

### 2.9 Outcome measures

For patients with an olecranon avulsion fracture in the anchor loop plate group, there is no need for brace fixation after surgery. On the second day after the operation, the dressing was opened to observe the wound, and the drainage tube was removed. Strictly disinfected the wound and replaced the dressing. X-ray and CT of the elbow joint were retrospectively analyzed. Moreover, encourage patients to start elbow flexion and extension movement and upper limb muscle contraction movement, the movement angle gradually increased, 4 weeks after surgery can start daily resistance exercises, 3 months to avoid strenuous exercise. During the first 3 months after the operation, an outpatient review was conducted once a month and then once every 3 months. The X-ray film of the elbow joint of the affected limb was reviewed to evaluate the fracture healing, and the patients continued to guide the elbow joint function exercise, record the elbow joint activity, and record the surgical complications. Fracture healing check was based on X-ray findings and clinical results. X-ray examination showed that the fracture was healing, with no fracture line. The clinical results showed that the patient could flex normally without elbow pain, and the fracture was healed. Mayo score (including elbow flexion/extension range, forearm rotation range, muscle strength and pain) (Cusick et al., 2014) and DASH score (referring to upper limb disease and upper limb function) (Gummesson et al., 2003) were used to evaluate functional recovery. The value range of both is 0–100. The higher the Mayo score, the better the results (95–100 is excellent, 80–94 is good, 60–79 is general, 0–50 is poor), while the higher the DASH score, the worse the results.

### 2.9 Statistical analysis

Data analysis was performed using SPSS version 22.0(SPSS Inc., Chicago, IL, United States). Continuity variables are represented by the mean ± standard deviation. Independent sample t-tests were used for comparisons between groups. *p* < 0.05 was considered to be statistically significant.

## 3 Results

### 3.1 Finite element analysis: displacement of fractures

**FIGURE 5:**
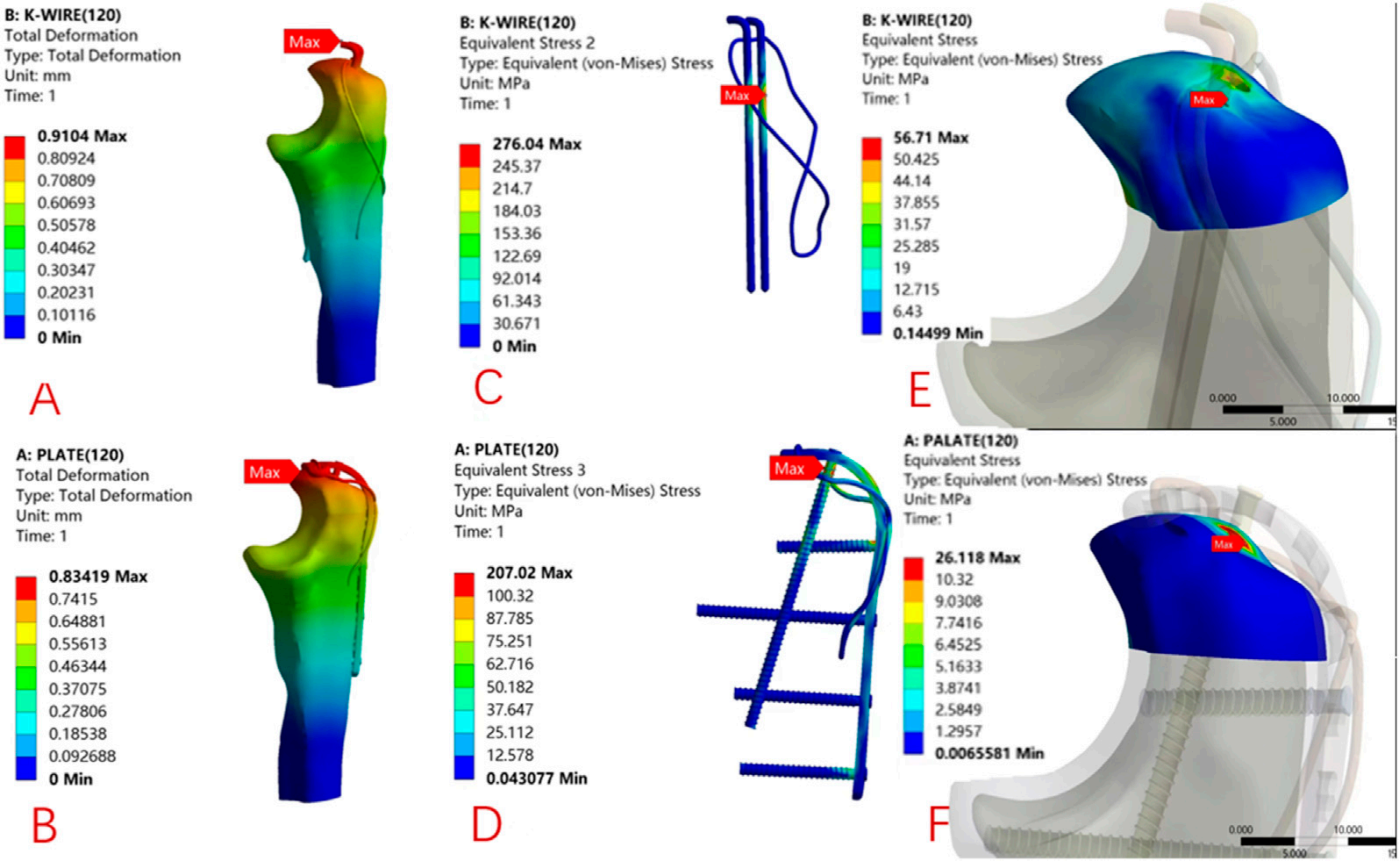
90° direction, 120 N load under the finite element analysis of cloud: K-wire group **(A)** and ALP group **(B)** model displacement distribution. The local stress distribution of internal fixation in the K-wire group **(C)** and ALP group **(D)**. The local stress distribution of the proximal olecranon fracture block in the K-wire group **(E)** and the ALP group **(F)** model.

**FIGURE 6:**
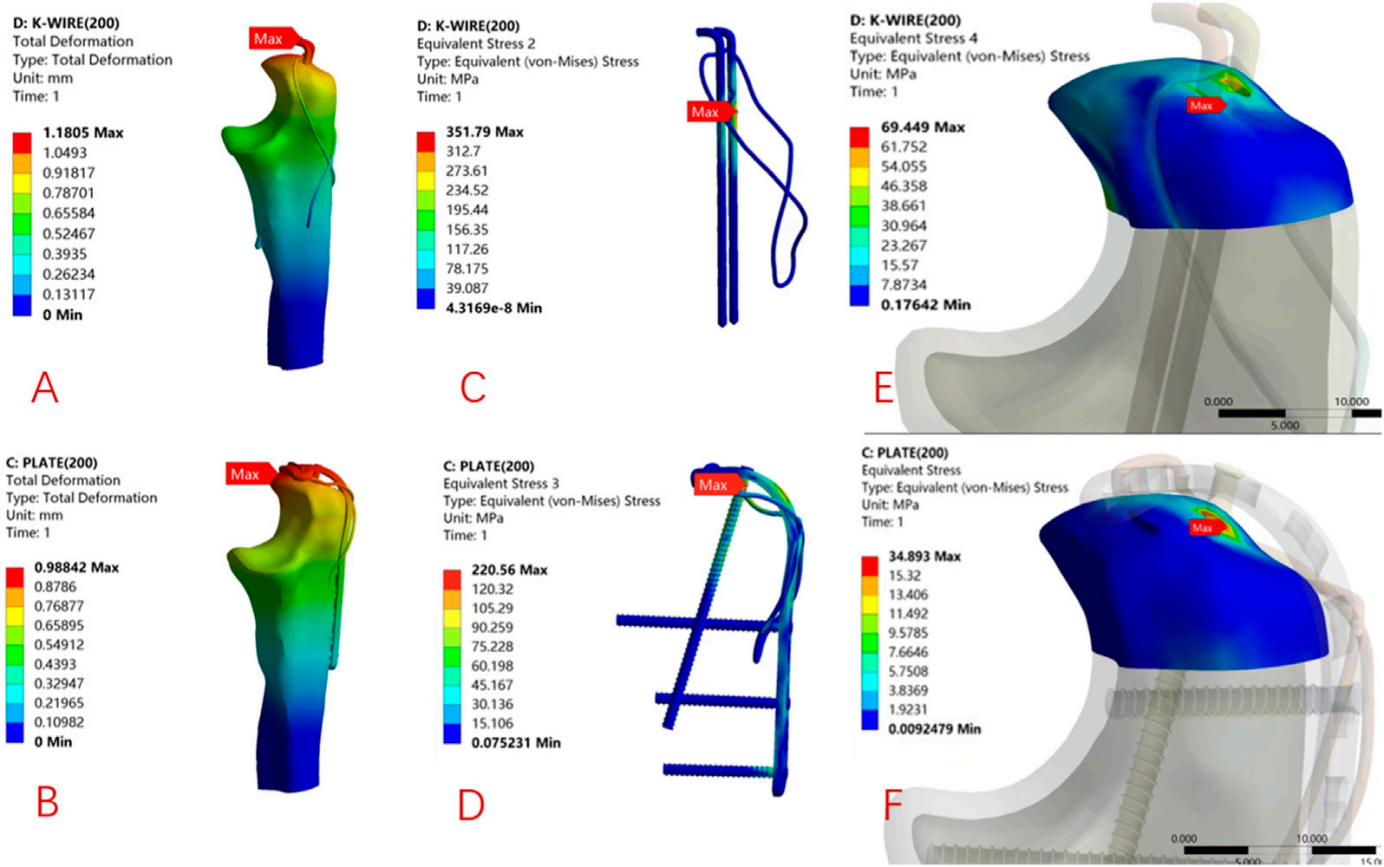
45° direction, 200 N load under the finite element analysis of cloud: K-wire group **(A)** and ALP group **(B)** model displacement distribution program. The stress distribution program of internal fixation in the K-wire group **(C)** and ALP group **(D)**. The stress distribution program of the proximal olecranon fracture block in the K-wire group **(E)** and the ALP group **(F)** model.

The biomechanical test results of the finite element model showed that the maximum displacements of the Kirschner wire group and the ALP group were 0.910 mm and 0.834 mm, respectively. The maximum displacement of the Kirschner wire group was 1.09 times that of the ALP group; the results of finite element analysis under 200 N load are as follows. The maximum displacements of the Kirschner wire group and ALP group were 1.181 mm and 0.988 mm, respectively. The maximum displacement of the Kirschner wire group was 1.19 times that of the ALP group, as shown in Figures 5, 6.

### 3.2 Finite element analysis: internal fixation stress and olecranon proximal stress distribution

Under 120 N load, the maximum internal fixation local stress of the K-wire and ALP groups were 276.04 MPa and 207.02 MPa, respectively. The maximum local stress of the Kirschner wire group was 1.33 times that of the ALP group. The maximum local stress of the proximal end of the olecranon in the K-wire and ALP groups was 56.71 MPa and 26.12 MPa, respectively. The maximum local stress of the proximal end of the olecranon in the Kirschner wire group was 2.17 times that of the ALP group. Under 200 N load, the maximum internal fixation local stress of the K-wire and ALP groups were 351.79 MPa and 220.56 MPa, respectively. The maximum local stress of the Kirschner wire group was 1.59 times that of the ALP group. The maximum local stress at the proximal end of the olecranon in the K-wire and ALP groups was 69.45 MPa and 34.89 MPa, respectively. The maximum local stress at the proximal end of the olecranon in the Kirschner wire group was 1.99 times that of the ALP group. The local stress is concentrated at the contact point of the internal fixation. The two sets of finite element analysis cloud pictures are shown in Figures 5, 6.

### 3.3 Clinical outcome

**FIGURE 7:**
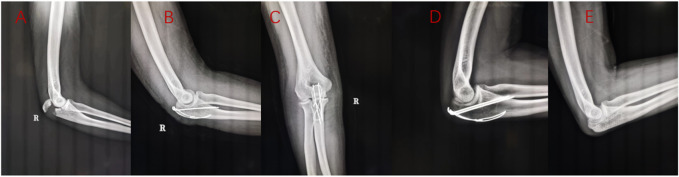
A 32-year-old female patient with right olecranon fracture caused by falling injury. Preoperative X-ray examination **(A)**, X-ray examination 2 days after Kirschner wire tension band fixation **(B, C)**, 3 months X-ray examination **(D)**,1 year later, the internal fixation was removed **(E)**.

**FIGURE 8:**
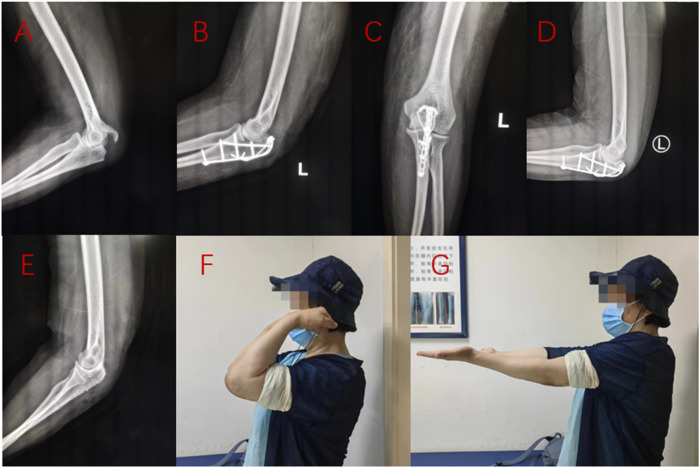
A 61-year-old female patient suffered from left olecranon fracture due to fall injury. Preoperative X-ray examination **(A)**, X-ray examination **(B, C)** 1 day after anchor fusion plate fixation, X-ray examination **(D)** 3 months after operation, X-ray examination **(E)** 1 year after internal fixation removal, and elbow function **(F, G)** 15 months after operation were performed.

A total of 53 eligible patients were included in this study. There were 31 cases in the Kirschner wire group, aged from 32 to 74 years old, with an average of 57.00 years old. The operation time was 82–126 min (mean, 108.52 min). The mean follow-up time was 15.87 months (14–20 months). The final elbow range of motion was 114°–127°, with an average of 119.97°. The Mayo score was 75–94 points, averaging 83.77 points. The DASH score was 16–26 points, averaging 20.97 points. The age of 22 patients in the ALP group ranged from 30 to 80, with an average of 60.27 years old. The operation time was 95–129 min (mean, 111.82 min). The mean follow-up time was 15.59 months (14–20 months). The final elbow range of motion was 119°–130°, with an average of 124.45°. The Mayo score was 81–97 points, with an average of 90.95 points. The DASH score was 12–24 points, averaging 17.36 points. The detailed statistical results are shown in Table 4. Typical working conditions are shown in Figures 7, 8.

**TABLE 4:** Comparisons of Kirschner wire tension band (K-wire) to Anchor loop plate (ALP) techniques for olecranon fracture (Mean ± SD).

Variables	K-wire (n = 31)	ALP (n = 22)	*p*-Value
Age (years)	57.00 ± 11.80	60.27 ± 13.00	0.345
Follow-up (months)	15.87 ± 1.69	15.59 ± 1.94	0.579
Duration of Surgery (min)	108.52 ± 12.41	111.82 ± 10.32	0.312
Final ROM	119.97 ± 4.11	124.45 ± 3.32	<0.05
Mayo score	83.77 ± 5.12	90.95 ± 5.18	<0.05
DASH score	20.97 ± 3.16	17.36 ± 3.93	<0.05

### 3.4 Complications

The X-ray examination 3 months after the operation showed that the olecranon bone healed, and the activity improved in the anchor loop plate and Kirschner wire groups. During the follow-up period, 1 patient had local soft tissue stimulation, and the internal fixation device was removed 1 year after the operation. There were no severe complications such as infection and reduction failure. In the Kirschner wire group, 5 patients had local soft tissue irritation, and the internal fixation device was removed after 1 year. One patient had an incision infection and underwent secondary debridement. The two groups’ specific complications and secondary surgery are shown in Table 5.

**TABLE 5:** Postoperative complications according to surgical technique.

*Technique*	*n*	*Infection n*	*Internal fixation stimulation n* (*%*)	*Reoperation n (%)*
K-wire	31	1 (3.2%)	5 (16.1%)	6 (19.0%)
ALP	22	$0\;\left(0\%\right)$	1 (4.5%)	1 (4.5%)
Total	53	1 (1.9%)	6 (11.3%)	7 (13.2%)

## 4 Discussion

### 4.1 Common internal fixation methods and defects of an olecranon fracture

The two most commonly used surgical options for treating olecranon fracture are the Kirschner wire tension band technique and the plate fixation technique (Metikala et al., 2021). The Kirschner wire tension band technique is more suitable for simple transverse fractures. Many previous studies have pointed out that the Kirschner wire tension band technique has achieved good clinical results in treating simple olecranon fractures and is a reliable surgical method (Chalidis et al., 2008; Huang et al., 2010). In addition, the implants used for tension band treatment are also cheaper. However, there have been many reports on the complications of the tension band technique. Because the Kirschner wire is partially in the medullary cavity will increase the risk of its proximal displacement; with the extension of internal fixation time, the Kirschner wire is prone to loosening, and the tail of the needle at the proximal end of the fracture is easy to form bursitis, and even puncture the skin to cause local infection (Wilkerson and Rosenwasser, 2014). The incidence of skin and soft tissue irritation caused by internal fixation can be as high as 40%–90%, the most common complication after tension band surgery (Carofino et al., 2007). In particular, internal fixation stimulation due to the proximal displacement of the Kirschner wire, of which about 60%–80% of patients must undergo secondary surgery to be able to remove the internal fixation to treat soft tissue irritation symptoms (Snoddy et al., 2014), resulting in additional financial burden. Other complications include nerve palsy and vascular injury, usually caused by migrating Kirschner wires to deeper planes (Parker et al., 2005; Rompen et al., 2010). Duckworth et al. (2017) compared the tension band technique with the plate fixation technique in 67 patients with acute olecranon fractures younger than 75. The results showed that the overall complication rate of the tension band technique was higher. To treat olecranon fractures, the need for secondary surgery to remove the internal fixator is still an essential disadvantage of the Kirschner wire tension band technique. Although the application of steel plate in the treatment of olecranon fracture also has the condition of internal fixation stimulating soft tissue, compared with the Kirschner wire tension band technique, the rate of postoperative reduction loss and the rate of secondary surgical removal of internal fixation are lower (Savvidou et al., 2020). However, the complications of avulsion fracture of triceps brachii after plate fixation are increasingly common (Orbay et al., 2022). Additionally enhanced suture is needed to improve the fixation strength and reduce the incidence of postoperative avulsion fracture complications. In our study, 5 cases (16.1%) in the Kirschner wire group had internal fixation irritation complications, and only 1 case (4.5%) in the anchor loop plate group had internal fixation irritation complications, all of which were removed by secondary surgery. No other postoperative complications occurred in the anchor loop plate. And the results of this study show that the use of ALP fixation technique in the treatment of olecranon fracture has better mechanical stability and better recovery of elbow joint function after operation. Therefore, ALP technique is an effective method for the treatment of olecranon fracture.

### 4.2 Biomechanical study of an olecranon fracture

Midtgaard et al. (Midtgaard et al., 2020) An *in vitro* mechanical study showed that there was no difference in fracture displacement between the tension band technique and plate fixation technique in transverse olecranon fracture, but in the evaluation of the mechanical failure of internal fixator, it was found that the steel wire cerclage structure of tension band was weak and prone to mechanical structure failure. Mechanical studies on olecranon fractures show that tension band technology is not the most stable fixation structure in treating olecranon fractures. A mechanical study on the fixation of olecranon fracture with a locking plate and intramedullary nail found that the fixation effect of the locking plate and the intramedullary nail was similar, both of which could stabilize the olecranon fracture (Nowak et al., 2013). Most previous studies were purely biomechanical studies, which clinical studies have not verified.

### 4.3 Clinical and biomechanical results of this study

Our finite element analysis shows that under the 120 N load in the 90° direction and the 200 N load in the 45° direction, the maximum displacement of the anchor loop plate is smaller, the stress of the internal fixator is more minor, and the fixation is more reliable, showing better biomechanical stability than the Kirschner wire tension band technology. The stress of the proximal olecranon fracture block in the anchor loop plate group was significantly smaller, and the complications of fracture block being cut by internal fixation and internal fixation failure were not easy to occur. There are many scales to evaluate the functional recovery of elbow joints after the operation. Among them, the Mayo and DASH scores are the most preferred and reliable scores, which are essential in evaluating the surgical effect of olecranon fracture (Çağlar et al., 2021). Our study used the Mayo and DASH scores to evaluate the recovery of elbow function after two internal fixation methods. The higher the Mayo score, the better the recovery of elbow function, and the higher the DASH score, the worse the recovery of elbow function. It was found that patients treated with anchor loop plates had higher Mayo scores and lower DASH scores. The elbow joint’s range of motion (ROM) in the anchor loop plate group was more extensive than that in the Kirschner wire tension band group 1 year after the operation, and the difference was statistically significant. The biomechanical stability of internal fixation is the basis of early functional exercise, but we should also pay attention to the bone mass condition of the fracture block itself. Our research shows that the local stress effect of the tension band on the bone block increases the risk of cutting, while the ALP has a larger contact area with the fracture block, which can effectively disperse the stress distribution between the internal fixation and the fracture block, and has better mechanical properties than the tension band. This is the basis of early functional exercise, which also explains why the ALP group can obtain higher scores earlier and shorter. In addition, by analyzing the collected clinical data, we noticed that the proportion of the elderly over 70 years old in the anchor loop plate group was 27.3%, while that in the Kirschner wire group was 6.5%. With the increase of age, osteoporosis is aggravated in patients, and there is more possibility of occult fracture on the fracture block, which makes the internal fixation more difficult. ALP, a combination of steel plate and steel wire, is similar to cage. The fracture block is placed in the cage and fixed in multiple directions. Combined with our clinical data, it is suggested that it has more advantages in patients with osteoporosis and comminuted fracture.

### 4.4 The potential advantages of anchor loop plate combined with steel wire binding technology compared with other internal fixation techniques

The reduction and fixation principle of the anchor loop plate is similar to that of the bridge fixation. The triceps tendon is dragged to the distal end of the olecranon at the connecting point of the olecranon bone to restore the continuity of the olecranon bone. At the same time, this anchoring reduction combined with the steel wire binding fixation method consists of the short arm of the proximal bending of the steel plate, the implanted screw, the “8’shaped steel wire and the surrounding soft tissue to form a structure like a” bird cage. This chooses surgery, whether it is a simple olecranon fracture, comminuted olecranon fracture, or avulsed olecranon fracture, the surgeon does not need to consider the size of the fracture block to replace the model of the plate, nor need to consider the severity of the crushing and use more plates to cover the comminuted fracture end. However, compared with the Kirschner wire tension band, the stress at the proximal end of the olecranon of the anchor loop plate is significantly smaller. This may be because the anchor loop plate structure has a larger contact area under the same load force, which makes the local pressure of the proximal fracture block of the olecranon smaller and less prone to the cutting of the fracture block by the internal fixation. An avulsion fracture occurs at the proximal end of the olecranon. When the fracture block is small, scholars generally believe the special olecranon plate for the proximal extension can better fix the proximal olecranon fracture fragments. However, it has obvious disadvantages: the internal fixation increases and the possibility of impact increases (Boden et al., 2019). The anchor loop plate we studied and designed cleverly solves this problem. The bending extension part is hidden under the construction point of the triceps brachii, which plays a good role in fixing the fracture block of the proximal avulsion and will not cause the apparent problem of local internal fixation. At the same time, the anchor loop plate we used is obtained by bending the common mini-plate, so the surgical method is easier to popularize. This kind of small steel plate is usually smaller, thinner and easy to bend, so its adhesion is more robust, and it is not easy to stimulate soft tissue internal fixation stimulation. Compared with Kirschner wire tension band technique, anchor loop plate is less prone to internal fixation failure and internal fixation soft tissue stimulation. Compared with the standard plate fixation technology, the anchor loop plate operation scheme has more straightforward requirements for the steel plate and does not need to reserve multiple types of steel plates. The smaller plate volume makes the internal fixation stimulation not easy to occur. In addition, the steel wire in the ALP structure retains the advantages of the tension band. Compared with the simple plate treatment, the ALP steel wire can provide better compression of the fracture block than the plate when it is tightened, and can penetrate the soft tissue in a similar way to the cerclage. The fracture fixation of the flanks on both sides of the complete plate is difficult to achieve by using the plate alone. Most importantly, the ALP’s fixation concept is to hang and fix the triceps tendon and the fracture block as a whole, so that the traction tension of the triceps tendon to the fracture block is dispersed on the ALP device. Compared with the previous concept of firmly fixing the fracture block and the fracture bed, the hang effectively solves the stress conduction between the triceps tendon and the ulna bone bed. Therefore, the risk of fracture block cutting failure is effectively avoided.

### 4.5 Conclusion and shortcomings of current research

Our study applied this surgical technique to clinical practice for the first time. Through the statistical analysis of clinical data, it was found that the elbow joint function of ALP group was better after operation. Therefore, we designed the FEA mechanics experiment. Through analysis, it was found that the ALP group had better biomechanical stability. In general, better biomechanical stability can support early functional exercise of the elbow joint in patients with olecranon fractures to obtain better elbow function. The experimental results of FEA in the mechanical stability of ALP support showed that patients in the ALP group could obtain better elbow joint function due to better biomechanical stability. Through the statistical analysis of clinical data and combined with FEA results. We get the following conclusions: In the treatment of olecranon fracture, compared with the traditional tension band, the ALP technique has better biomechanical stability and better recovery of elbow joint function after the operation. The ALP technique is a more effective surgical scheme for treating olecranon fractures. The disadvantage is that the sample size included in this study is small, and a larger sample size is needed to verify the clinical effect of this new surgical technique. The biomechanical model of finite element analysis is simplified, ignoring the influence of soft tissues, such as ligaments and muscles around the elbow joint, on the mechanical stability, which may cause specific errors. Further mechanical experiments are needed to verify our research results.

## Data Availability

The datasets presented in this study can be found in online repositories. The names of the repository/repositories and accession number(s) can be found in the article/Supplementary Material.
